# Mental health implications on Afghan children: an impending catastrophe

**DOI:** 10.1017/gmh.2022.43

**Published:** 2022-08-18

**Authors:** Khulud Qamar, Priya, Aiman Rija, Laiba Imran Vohra, Faisal A. Nawaz, Mohammad Yasir Essar

**Affiliations:** 1 Dow Medical College, Dow University of Health Sciences, Karachi, Pakistan; 2 Ziauddin University, Karachi, Pakistan; 3 College of Medicine, Mohammed Bin Rashid University of Medicine and Health Sciences, Dubai, United Arab Emirates; 4 Kabul University of Medical Sciences, Kabul, Afghanistan

**Keywords:** Afghanistan, awareness, children, humanitarian crisis, mental health

## Abstract

Afghan children have suffered for decades because of chronic socioeconomic health crises. The current state of Afghanistan has deprived the basic human needs of children. The lack of freedom leaves their voices unheard, causing detrimental effects on their mental health. Mental illnesses such as post-traumatic stress disorder, anxiety, and depression are prevalent in Afghanistan, causing severe negative outcomes among children. Promotion of mental health services, psychological training, awareness campaigns, acceptance of Afghan refugees, and initiatives to support re-connecting with loved ones, are among the many recommended measures needed to manage this alarming situation. This requires an immediate action plan from government and public health officials to mitigate this impending catastrophe.

## Introduction

The Taliban have taken over the constitutional authority of Afghanistan in 2021 after two decades of US-led invasion. The current Taliban leadership has assured the presence of ‘lasting peace, prosperity and development’ for the country. However, many citizens have been restricted from attaining education, contacting media personnel, government, or military services, as well as receiving physical and mental health services (Al Jazeera, n.d.-b). While Afghanistan is now considered an independent nation, it has proven challenging to maintain viability due to the poor state of the nation (Essar *et al*., 2021). Despite monetary aid being regulated by welfare organizations and the USA, amounting to over 8 billion US dollars since 2021, an economic crisis still prevails with an urgent need to address food insecurity, shelter, clean water, and sanitization (Save the Children's Response in Afghanistan, 2022). The long-term challenge of political corruption has led to financial insecurity, as around 97% of Afghan people continue to struggle in poverty. Most citizens are unable to afford basic food and water supplies, health services, and other vital necessities (Al Jazeera, n.d.-a). The United Nations has reported that 9 million people are currently suffering from food shortages in the country. To make ends meet, children pursue forced labor instead of attending school. Child labor may include working at construction sites, cleaning facilities, and at times being exploited into sex work as well. It is also reported civilians have taken drastic measures such as selling their unborn babies to make ends meet (Islam *et al*., 2021; Billing Lynzy, 2022). The safety of the people living in Afghanistan is compromised with rising violence rates, making children and youth prone to high rates of substance abuse, possession of weaponry, and exposure to criminal activities.

During such challenging times, children are most vulnerable due to lack of independence, and are often left helpless in the midst of extreme hardships (Trani *et al*., 2013). The lack of freedom and choice often stem from poverty coupled with social and cultural pressures within society. Male children are forcefully sent to work by their families as the sole breadwinners. Failure to live up to this responsibility is seen as a sign of dishonor in the family (Eggerman and Panter-Brick, 2010). Female children are forced to marry or work as maids in homes; refusal to do so creates the impression of financial burden within their families (Eggerman and Panter-Brick, 2010). Children also suffer from acute malnutrition, physical harm, drug abuse, and lack of healthcare services (Trani *et al*., 2013). Malnutrition among Afghan children has affected their growth and has resulted in mineral deficiencies and other illnesses. Additionally, children are easily targeted in gun shootings, gang fights, abductions, kidnappings, and sexual abuse. Substance abuse is quite common among adults, which translates to unsupervised consumption of drugs among children, thus increasing the risk of drug overdose in the pediatric population. These conditions severely affect mental well-being of children, as support services including child protection are nearly non-existent (Shoib *et al*., 2022). Of all the functioning medical centers, less than 1% of them offer mental health support. The ratio of health care workers to patients has been less than 10 per 10 000 people (Islam *et al*., 2022). Collectively, this existing burden hampers any steps toward strategizing existing resources. The overall impact of worsening child mental health will massively demote Afghanistan's progress as children play a crucial role in shaping the future community needs.

## Challenges to child mental health in Afghanistan

Hageman *et al*. stated in one of their studies that Afghanistan has historically been one of the most hazardous places on the planet for children (Hageman and Alkureishi, 2021). The burden of poor mental health originates in Afghanistan because of widespread poverty, social inequity, and recurrent wars. Additionally, social expectations and cultural norms contribute to create unjust barriers for Afghans due to which the ability to attain personal and societal goals influences the national mental well-being in a negative manner. Afghan people, including children, rely on strong interpersonal beliefs to face societal challenges. This is also reflected in a communal sense of faith in God by practicing resilience and patience during adversities. Individuals who seek mental help are disregarded by Afghan society as they are considered ‘crazy’ or ominous. This further brings them into religious and cultural entrapment, and prevents vulnerable citizens from recognizing and seeking care for their mental health disorders (Eggerman and Panter-Brick, 2010).

Eggerman *et al*. have mentioned in their observational study that economic problems have immensely impacted the younger age groups. Parents associate eradication of poverty with achieving high grades in schools (Eggerman and Panter-Brick, 2010), which creates a vicious cycle of socioeconomic stress in academia and adds to the children's frustration. Due to the economic instability of their families, one in every five children has reported working to earn money while attending school. Children have expressed strong anxieties toward the possible socioeconomic outcomes if the breadwinner of the family is lost to an illness or a disability. In addition to such difficulties, 40% of Afghans believe there is no way out from this prevailing humanitarian crisis (Eggerman and Panter-Brick, 2010).

Therefore, because of the humanitarian crises, many Afghans have lost their homes, possessions, and their loved ones (War Child, n.d.). Lack of certainty about their future and distress about death have resulted in widespread mental health issues (Shoib *et al*., 2022). Children are especially prone to being victimized by persistent violence and they suffer from both immediate and long-term psychological consequences (Shoib *et al*., 2022). According to a 2018 cross-sectional study on the children of Afghanistan, 71% experienced physical violence in the previous year. Afghan children are raised under persistent threats of violence and are subjected to a plethora of human right atrocities such as child marriages, physical assault, and labor exploitation (War Child, n.d.). Children who are subjected to violence, child abuse, or social and emotional aggression are at a high risk of developing cognitive and social difficulties that can last into adulthood or lead to worsening mental health conditions (Neto *et al*., 2022). According to recent studies, more than one-third of youngsters have experienced psychological trauma as a result of the loss of peers, family, continual death risk, and injury (Kovess-Masfety *et al*., 2021). Furthermore, the onset of COVID-19 alongside present problems has burdened mental health demands by creating an additional fear of death (Essar *et al*., 2021). The aforementioned data essentially show that apparent vulnerability, anxiety, assault, agony, and psychological discomfort all have a detrimental impact on the mental health and neurocognitive development in children (Kovess-Masfety *et al*., 2021). Since the Taliban takeover, instability has reached to its peak, as 9 million Afghans have been reportedly displaced, and 1 million children are said to be severely malnourished (Hannah, 2021; UNHCR, 2022). The political conflict and instability led to many residents fleeing the country resulting in separation from their loved ones, which further impacted the mental health of Afghan citizens (Von Werthern *et al*., 2019).

Children are thought to be more susceptible to such illnesses due to their need for dependency and care. As a result, their psychological symptoms often persist throughout their lives (Naz, 2021). Afghan children begin to face the brunt of psychological distress the moment they are born into a country experiencing uncertainty amidst conflict for decades (Islam *et al*., 2022). The absence of a healthy and stable environment while growing up, and exposure to trauma, predisposes Afghan children to a state of chronically declining mental health (Naz, 2021). Recent studies portray that more than a third of children face distress of some kind, which further details the impact of loss and fear of death on child psyche (Nascimento *et al*., 2022).

Individuals who have witnessed violence or felt threatened by a traumatic event in their lives are at risk of developing post-traumatic stress disorder (PTSD) (Torres, 2020). The prevalence of PTSD among Afghan children can be clearly identified through a cross-sectional study showing 42% of refugee minors from Afghanistan experiencing symptoms of PTSD – the highest among all the countries included in the study (Solberg *et al*., 2020). In a survey including 600 Afghan parents, 73% of parents reported that their children have experienced feelings of anxiety and depression (Afghanistan: Many Afghan Children Are Afraid to Go Outside, New Survey by Save the Children Finds, 2019). Children fear stepping out of their homes due to perceived threats of explosions, kidnappings, and gunshots (Afghanistan: Many Afghan Children Are Afraid to Go Outside, New Survey by Save the Children Finds, 2019). This may cause them to feel threatened even in the absence of visible danger. Furthermore, 38% of parents reported their children facing a wide variety of mental health struggles ranging from insomnia and prolonged sadness, to attempts of self-harm (Afghanistan: Many Afghan Children Are Afraid to Go Outside, New Survey by Save the Children Finds, 2019).

The turmoil and poverty in Afghanistan impose major obstacles in achieving optimal physical and psychological development of the Afghan children (Naz, 2021). Since children find it particularly challenging to face such stressors, their current state is not only linked to a mental health diagnoses, but also adverse childhood effects (ACEs) (Jones *et al*., 2020). This includes difficulty in maintaining family and peer relationships, school performance, ability to adapt, and general life satisfaction. Children tend to internalize this trauma if they do not find a caregiver who can effectively regulate their emotions (Catani, 2018). In an attempt to manage high stress levels and uncomfortable emotions, children may resort to unhealthy coping mechanisms and bad lifestyle choices. In the long-term period, both their physical and mental health are severely affected, as adults with ACEs tend to suffer with depression, obesity, and cardiovascular diseases. This also includes the growing statistics of drug addiction among children in Afghanistan (Hadid and Ghani, 2019).

Overall, this demonstrates the immediate need for necessary mental health interventions to be made for children living in conflict-afflicted areas like Afghanistan.

## Recommendations

To improve the mental health of children in Afghanistan during this humanitarian crisis, non-medical professionals should be trained to convey basic counseling (Shoib *et al*., 2022). A randomized controlled trial in Nepal revealed that non-medical health worker-led counseling of patients attending primary care led to a 50% reduction of perceived stress in the Beck Depression Inventory (BDI) score. The training consisted of components focused on theoretical background, basic therapeutic skills, components of cognitive-behavioral therapy, problem-solving, exposure therapy, yoga, and meditation (Markkula *et al*., 2019). Further psychotherapeutic and relevant novel therapies should be done to avoid subsequent trauma (Shoib *et al*., 2022). For example, trauma-focused treatments such as prolonged exposure and cognitive processing therapy, and non-trauma-focused treatments such as relaxation, stress inoculation training, and interpersonal therapy should be implemented to reduce PTSD symptoms (Watkins *et al*., 2018). The programs would be delivered in an individual or group setting, including a variable number of sessions, with interventions such as psychoeducation, stretching, guided imagery, and meditation (Kananian *et al*., 2021). However, a shortage of qualified psychiatrists, psychiatric nurses, psychologists, and social workers can pose a significant quality of care problem for the provision of such mental health services (PTSD in Afghanistan: Burden, Challenges and Ways Forward, LLU Institute for Health Policy Leadership, n.d.). It is important for children to connect with parents and peers to find natural ways to regulate their emotions. Child-friendly environments and operation for helplines should be offered, in addition to providing monetary assistance to families in order to provide supplemental earnings and guarantee that their children receive optimal education (War Child, 2022). UNICEF has had a field presence in Afghanistan for much more than half a century, and assists in multiple areas such as establishing nutritional hubs and vaccination centers, helping students in community-based education curricula, along with a $192 million appeal to meet the ever-increasing refugee catastrophe (UN News, 2021). The optimal recommendation for improving mental well-being is the normalization of living circumstances and the alleviation of socioeconomic problems resulting from poverty and insecure livelihoods. This calls for the development of community programs to promote income generation which will have direct beneficial effects on children and their families (Ventevogel *et al*., 2013).

Furthermore, all nations should be prepared to accept Afghan refugees, and guarantee that Afghan children and families have access to crucial services (UNICEF, n.d.). Campaigning for womens' and girls' right to emotional, physical, and mental healthcare should continue by preventing social discrimination early on and avoiding the development of depression and other mental health disorders that are a result of social confinement (Muslim Aid, 2020). An additional approach is the promotion of ‘child-centered spaces’, where children struggling with traumatic experiences can re-socialize through play and education in the norms and values of the peaceful Afghan society, such as the Consortium for the Psychosocial Care and Protection of Children, formed by three major international NGOs (Child Fund Afghanistan, Save the Children USA and International Rescue Committee) with the aim to improve the psychosocial well-being and development opportunities for Afghan children (Ventevogel *et al*., 2013). Grave breaches in child rights must be strictly condemned so that this vulnerable population does not bear the brunt of violence, instability, and economic turmoil in the region. Further research is called for in exploring the trends and impact of positive measures in guiding future mental health policies for Afghan children.

## Conclusion

In conclusion, the ongoing socioeconomic unrest, combined with the COVID-19 pandemic, food insecurities, inaccessibility of essential resources such as sanitation and shelter, lack of healthcare services, and decreased opportunities to literacy, is creating a mental health catastrophe for the children of Afghanistan. As a result, Afghan children have become increasingly vulnerable to mental health disorders. Selective interventions such as providing basic health services and security, community support, and specialized training programs, could help ameliorate the mental health challenges faced by children. Further research is warranted to understand the influence of conflict on children in order to devise timely interventions for all age groups.
